# Allicin Improves Metabolism in High-Fat Diet-Induced Obese Mice by Modulating the Gut Microbiota

**DOI:** 10.3390/nu11122909

**Published:** 2019-12-02

**Authors:** Xin’e Shi, Xiaomin Zhou, Xinyi Chu, Jie Wang, Baocai Xie, Jing Ge, Yuan Guo, Xiao Li, Gongshe Yang

**Affiliations:** Key Laboratory of Animal Genetics, Breeding and Reproduction of Shaanxi Province, Laboratory of Animal Fat Deposition and Muscle Development, College of Animal Science and Technology, Northwest A&F University, Yangling 712100, China; xineshi@nwafu.edu.cn (X.S.); xmzhou112@126.com (X.Z.); chuxy0919@163.com (X.C.); wangjie93@nwafu.edu.cn (J.W.); xiebaocai2017@nwafu.edu.cn (B.X.); jingjing1991@nwafu.edu.cn (J.G.); nice.lixiao@gmail.com (X.L.)

**Keywords:** allicin, gut microbiota, obesity, adipose, intestinal microbiota

## Abstract

Allicin, naturally present in the bulbs of the lily family, has anticancer, blood pressure lowering, blood fat lowering and diabetes improving effects. Recent studies have shown that allicin promotes the browning of white adipocytes and reduces the weight gain of mice induced by high-fat diet. While the gut microbiota has a strong relationship with obesity and energy metabolism, the effect of allicin on weight loss via gut microorganisms is still unclear. In this study, we treated obese mice induced by high-fat diet with allicin to determine its effects on fat deposition, blood metabolic parameters and intestinal morphology. Furthermore, we used high-throughput sequencing on a MiSeq Illumina platform to determine the gut microorganisms’ species. We found that allicin significantly reduced the weight gain of obese mice by promoting lipolysis and thermogenesis, as well as blood metabolism and intestinal morphology, and suppressing hepatic lipid synthesis and transport. In addition, allicin changed the composition of the intestinal microbiota and increased the proportion of beneficial bacteria. In conclusion, our study showed that allicin improves metabolism in high-fat induced obese mice by modulating the gut microbiota. Our findings provide a theoretical basis for further elucidation of the weight loss mechanism of allicin.

## 1. Introduction

The incidence of overweight and obesity is increasing around the world. Epidemiological studies have identified a high body mass index as a risk factor for a range of chronic diseases, including cardiovascular disease, type 2 diabetes, chronic kidney disease, multiple cancers, and a range of musculoskeletal disorders [1,2]. The prevention of obesity has become among the most challenging concerns for modern society. A growing amount of evidences suggest that the gut microbiota may serve as an important modulator of obesity by affecting the absorption of nutrients in the intestine [3,4].

Recent studies have shown that gut microorganisms function similarly to endocrine organs, because they produce biologically active metabolites that affect the host. Intestinal microorganisms are in a position to produce large amounts of metabolites—some of which are absorbed directly into the systemic circulation and others are processed by the host enzymes [5]. The intestinal microbiota of healthy people is mainly composed of Bacteroidetes and Firmicutes. It has been found that in obese individuals, the abundance of Bacteroidetes is decreased and the abundance of Firmicutes is increased [6]. This suggests that Bacteroidetes and Firmicutes are essential for regulating obesity and can serve as obesity markers.

Allicin and its derivative, diallyl disulfide (DADS), are trithioallyl ether compounds that naturally occur in the bulbs of the lily family member, garlic, and are produced by alliinase, which produces allicin [7]. Allicin is highly reactive with thiol groups and, under certain conditions, it also reacts with itself, forming further compounds that are also bioactive, such as vinyl-dithiins, ajoene and polysulfanes [8]. Furthermore, allicin is an active sulfur (RSS) with oxidative properties that can regulate oxidative metabolism in cells. [9].

Allicin’s structure determines many of its biological functions. For example, Feldberg et al. have found that allicin affects the synthesis of macromolecules such as DNA, RNA and protein [10], and garlic consumption or the inclusion of garlic oils in the diet selectively reduces the concentration of blood triglycerides (TGs), total cholesterol (TC) and low-density lipoprotein-cholesterol (LDL-C) without affecting high-density lipoprotein-cholesterol (HDL-C) levels [11,12]. The latest research has found that allicin promotes white adipose tissue browning through krüppel-like factor (KLF)15 [13]. Some studies have also found that allicin has several biological functions, including antibacterial, blood pressure lowering and antioxidation [14,15,16]. In addition, DADS and diallyl polysulfone, directly formed by the decomposition of allicin, have exhibited remarkable antibacterial effects. Koch and Lawson have demonstrated that allicin inhibits the growth of *Escherichia coli* and *Staphylococcus aureus* [17]. These studies indicate that allicin affects both fat deposition and microorganisms. Hence, we wondered whether these physiological functions of allicin are achieved by regulating the gut microbiota. However, we could not find data about allicin regulating the gut microbiota.

In this study, we explored the potential effects of allicin on mice with high-fat diet-induced obesity. We found that allicin suppressed body weight gain by regulating the gut microbiota.

## 2. Materials and Methods

### 2.1. Animal Experiment

Six-week-old C57BL/6 male mice, weaned from 4 weeks, were purchased from the Medical Laboratory Animal Center of Xi’an Jiaotong University (Xi’an, China; approval XJTULAC-2013-024). The animals were housed in stainless steel cages at room temperature (25 ± 2 °C), with a 12 h light/dark cycle. They were fed a commercial chow for a week to acclimatize to animal facilities and then weighed and randomly divided into two groups. One group was fed regular chow (control group, NFD, *n* = 6) and the other group received a high-fat diet (HFD, *n* = 12). We began the experiments once there was a significant difference in body weight between the HFD and NFD groups. The HFD group was randomly divided into two groups, which continued to receive a high-fat diet: one group was given normal saline (negative control, NC) and the other group was given 100 mg/kg/d allicin (Allicin) (S25256, Source Leaf Biological, Shanghai, China). The HFD in this study contained 60% fat and the NFD contained 10% fat (TrophicDiet, Nantong, China). During the experiments, body weight and feed intake were measured weekly. Mice were fasted overnight before being sacrificed; body weight was measured and tissues (inguinal white adipose tissue (iWAT), epididymal WAT (eWAT), brown adipose tissue (BAT) and liver) were excised, weighed and stored at −80 °C. In addition, the small intestine was immediately ligatured, and the contents were collected under aseptic conditions and frozen in liquid nitrogen for 16S rDNA sequencing. All animal procedures were performed in accordance with the Guidelines for Care and Use of Laboratory Animals of Northwest A&F University and were approved by the Animal Ethics Committee of Northwest A&F University (approval number NWAFU-314020038). The animal experiments were confirmed by the Guide for the Care and Use of Laboratory Animals of China.

### 2.2. Glucose Tolerance Tests

After six weeks of allicin administration, obese mice were fasted overnight. Tail vein blood was used to measure glucose levels using a YUWELL 560 glucometer (Jiangsu, China). Glucose levels were measured twice at every time point (0, 15, 30, 60 and 120 min) after intraperitoneal injection of 1 g of glucose (Cat. No. XK 13-201-00310, Tianjin, China) per kg body weight dissolved in saline.

### 2.3. Serum Analysis

The mice were treated with ether and the heart blood was collected and centrifuged at 13,680× *g* for 10 min. The collected serum was used to determine the concentrations of serum cholesterol (TC), serum triglycerides (TG), high-density lipoprotein (HDL), low-density lipoprotein (LDL), aspartate amino transaminase (AST) and alanine amino transaminase (ALT).

### 2.4. Haematoxylin and Eosin (H&E) Staining

The iWAT, eWAT, BAT and small intestine tissue from representative mice of each group were fixed with 4% paraformaldehyde. After samples were dehydrated and embedded in paraffin, sections were cut using a Leica RM22559 microtome (Leica, Shanghai, China) and standard H&E staining was performed.

### 2.5. PCR Real-Time Quantitative PCR (RT-qPCR)

Total RNA was extracted using TRIzol reagent (TaKaRa, Otsu, Japan) following the manufacturer’s instructions. The mRNA was reverse transcribed with transcription kits (TaKaRa) to synthesise cDNA, and the cDNA was amplified using SYBR Green kits on a Stepone plus™ system (Thermo Fisher, Waltham, MA, USA). The primer sequences used are shown in Supplementary Table S1, and the data were processed using the 2^−ΔΔ*C*T^ method.

### 2.6. Enzyme Activity Evaluation

The intestinal content of the enzymes, trypsin, amylase and lipase was determined by enzymatic activity kits (Institute of Bioengineering, Nanjing, China), according to the manufacturer’s instructions.

### 2.7. 16S rRNA Sequencing with Illumina MiSeq Sequencing

DNA was extracted from the intestinal contents using the Qiagen QIAamp DNA Stool Mini kit (Qiagen, Hilden, Germany) according to the manufacturer’s directions. Illumina MiSeq sequencing and general data analyses were performed by Novogene, Beijing, China. The DNA (regions V3 and V4 of the bacterial 16S rRNA gene) was amplified with barcoded specific bacterial primers using PCR. The primers used were 338F: 5′-ATCCTACGGGAGGCAGCA-3′ and 806R: 5′-ggactachvgggtwtctaat-3′. Amplification was performed with the DNA template (50 ng) in a 25 μL reaction for 25–35 cycles with Phusion DNA Polymerase.

### 2.8. Bioinformatic Analysis

MiSeq sequencing results in double-ended sequence data. First, we filtered the measured fq data, then we filtered bases with a read-tail mass value of 20 or less, and then set a 50 bp window. If the average mass value in the window was lower than 20, the window began to intercept the back-end base and filtered the read below 50 bp after the quality control; then, the paired sequences were merged into a sequence according to the overlap relationship of the Paired-End (PE) sequencing. Then, the sequences were grouped into operational taxonomy units (OTUs) at 97% similarity. Basically, there was less than 3% sequence dissimilarity in all reads of the same OTU. The Ribosomal Database Project (RPD) was applied to classify the OTU sequences and identify the bacterial species.

### 2.9. Statistical Analysis

All data are expressed as the mean ± SD, and statistical analysis was performed with GraphPad Prism 7.0. Data were analyzed by Student’s t-test. *p* < 0.05 was considered statistically significant, and *p* < 0.01 was considered highly statistically significant (* *p* < 0.05; ** *p* < 0.01).

## 3. Results

### 3.1. Allicin Reduces Body Weight Gain and Fat Deposition in a Mouse High-Fat Diet-Induced Obesity Model

**Figure 1:**
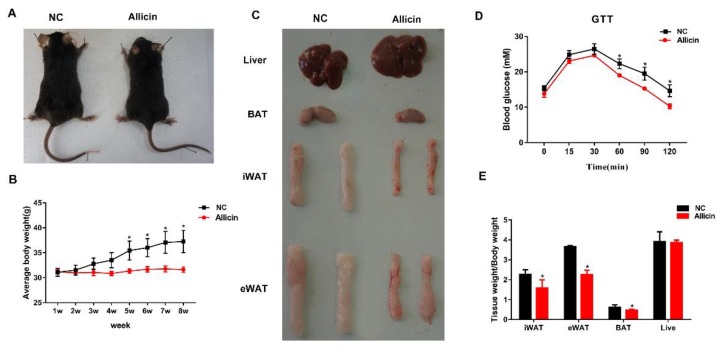
Allicin reduced body weight and fat deposition in mice with high-fat diet-induced obesity. (**A**) Image of mouse body. (**B**) Body weight over time. (**C**) Representative images of liver and adipose tissues. (**D**) Glucose tolerance test (GTT) results. (**E**) Adipose tissues and liver index (tissue weight divided by body weight). NC: high-fat diet group; Allicin: HFD plus allicin (100 mg/kg/d) group. The values represent the mean ± SD. * *p* < 0.05 and ** *p* < 0.01; *n* = 6. Note: NC is a negative control group—only oral normal saline for obese mice.

To investigate the effect of allicin on body weight and fat deposition, we established an obese mouse model by feeding mice a high-fat diet. Allicin or normal saline was orally administered to the obese mice. Compared with the NC group, the Allicin group had significantly decreased fat mass after 8 weeks (Figure 1A,C). The body weight and food intake were measured every week and there was a significant difference between the NC group and the Allicin group; the Allicin group gained less body weight compared with the NC group (*p* < 0.05; Figure 1B). However, there was no difference in food intake (data not shown). The glucose tolerance test (GTT) revealed that allicin significantly improved glucose tolerance (*p* < 0.05; Figure 1D). After the mice were sacrificed, fat and liver composition was analyzed. We found that the weight of adipose tissue from mice treated with allicin was significantly lower than that from the NC group, but the liver weight did not change (*p* < 0.05; Figure 1E). These results indicated that allicin reduced body weight and fat in mice with high-fat diet-induced obesity.

### 3.2. Allicin Ameliorates Blood Metabolic Parameters in Mice with High-Fat Diet-Induced Obesity

Studies have found that obesity is often accompanied by high serum cholesterol (TC) and TG [18], high TG and low HDL-C associated with insulin resistance, and several metabolic diseases [19]. To investigate whether allicin improves abnormalities in serum metabolic parameters in obese mice, we analyzed the concentrations of serum TC, total TG, HDL-C and LDL-C (Table 1). The results showed that allicin significantly increased the serum TC and HDL-C levels (*p* < 0.05), and decreased the serum LDL-C level, though not significantly (*p* > 0.05), whereas it did not affect the serum TG level. ALT and AST are used to assess liver damage by drugs; to determine the effect of allicin on the liver, we measured serum AST and ALT levels. After treatment with allicin, these parameters remained at their normal levels. These results indicated that allicin significantly improved blood metabolic parameters in obese mice induced by high-fat diet.

### 3.3. Allicin Reduced Lipid Droplets and Increased the Expression of Genes Involved in Lipid Metabolism in Obese Mice Induced by High-Fat Diet

**Figure 2:**
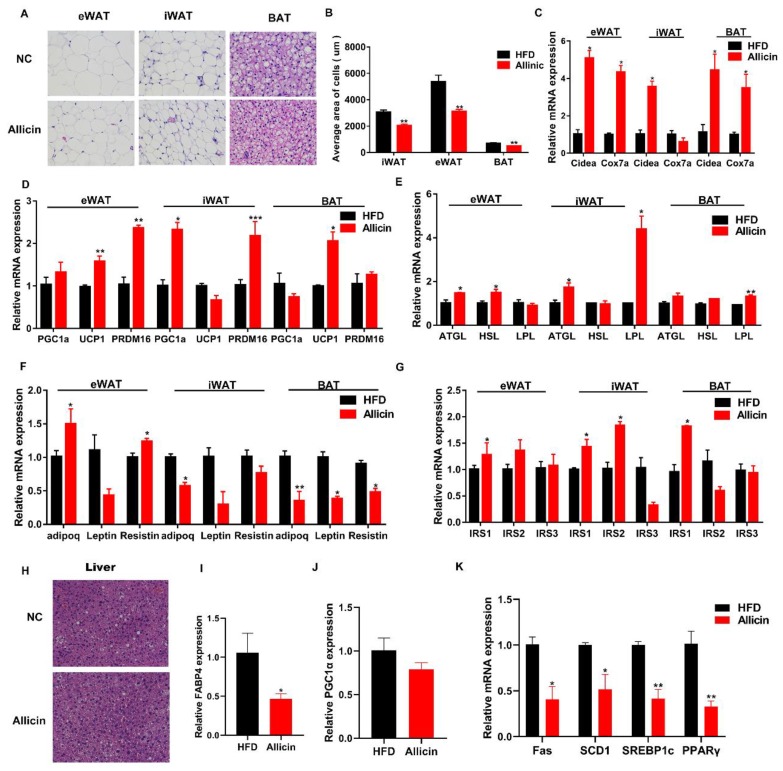
Allicin reduced lipid droplets and increased the expression of genes involved in lipid metabolism in obese mice induced by high-fat diet. (**A**) Haematoxylin and Eosin (H&E) staining of inguinal white adipose tissue (iWAT), epididymal WAT (eWAT) and brown adipose tissue (BAT) liver from representative mice of each group. Images are shown at the original magnification of 100×. (**B**) The mean cell area in iWAT, eWAT and BAT of each group (*n* = 3). (**C**–**G**) RT-qPCR results of the mitochondria-related genes, *Cidea* and *Cox7a*, the lipolysis-related genes, *ATGL*, *HSL* and *LPL*, the thermogenesis-related genes, *PGC1α*, *UCP1* and *PRDM16*, the adipokine-related genes, *leptin*, *adipoq* and *resistin*, and the insulin signaling pathway-related genes, *IRS1*, *IRS2* and *IRS3*, in iWAT, eWAT and BAT. (**H**) H&E staining of liver tissues from representative mice of each group. (**I**–**K**) RT-qPCR results of the lipid transport-related gene, FABP4, the lipogenesis genes, *FAS*, *SCD1*, *SREBP1c* and *PPARγ*, and the lipogenesis gene, *PGC1α*. Images are shown at the original magnification of 100×. The values represent the mean ± SD. * *p* < 0.05, ** *p* < 0.01 and *** *p* < 0.001; *n* = 6. Scale bar is 200 μm.

In obesity, lipid droplets become larger, the space between cells becomes smaller and the surrounding cells are squeezed. Moreover, lipolysis and heat production are reduced, causing inflammation and insulin resistance [20]. To determine the effects of allicin on the lipid metabolism of HFD mice, adipose tissue samples were prepared and stained with H&E. As shown in Figure 2A,B, compared with the NC group, iWAT, eWAT and BAT adipocytes were smaller in the Allicin group, with a significantly decreased average cell area (*p* < 0.01; Figure 2B).

Fat deposition is closely associated with the expression level of genes involved in lipogenesis and metabolism. To further elucidate the effect of allicin on lipid droplet deposition, using RT-qPCR, we examined the expression levels of the mitochondria-related genes, *Cidea* and *Cox7a* (Figure 2C), the lipolysis-related genes, *ATGL*, *HSL* and *LPL* (Figure 2D), the thermogenesis-related genes, *PGC1α*, *UCP1* and *PRDM16* (Figure 2E), the adipokine-related genes, leptin, adipoq and resistin (Figure 2F), and the insulin signaling pathway-related genes, *IRS1*, *IRS2* and *IRS3* (Figure 2G). The expression levels of lipolysis, mitochondria, thermogenesis and insulin signaling pathway-related genes were increased upon allicin treatment. In contrast, the adipokine-related genes were decreased after allicin treatment in the three adipose tissues tested.

The liver acts as the metabolic center of the body and is the main site for lipid production. Obesity causes non-alcoholic fatty liver and other related diseases. As shown in Figure 2H, compared with the NC group, allicin supplementation dramatically suppressed lipid accumulation in the liver. Furthermore, allicin suppressed the transcriptional expression of the lipid transport gene, *FABP4* (*p* < 0.05; Figure 2I), and the lipogenesis genes, *FAS*, *SCD1*, *SREBP1c* and *PPARγ* (*p* < 0.05; Figure 2K). Interestingly, allicin supplementation did not change the mRNA expression of the lipolysis gene, *PGC1α* (Figure 2J). These results indicated that allicin improved lipolysis, heat production and insulin sensitivity in mice with high-fat diet by suppressing hepatic lipid synthesis and increasing adipose tissue lipolysis and thermogenesis.

### 3.4. Allicin Improves the Intestinal Morphology of Obese Mice Induced by High-Fat Diet

**Figure 3:**
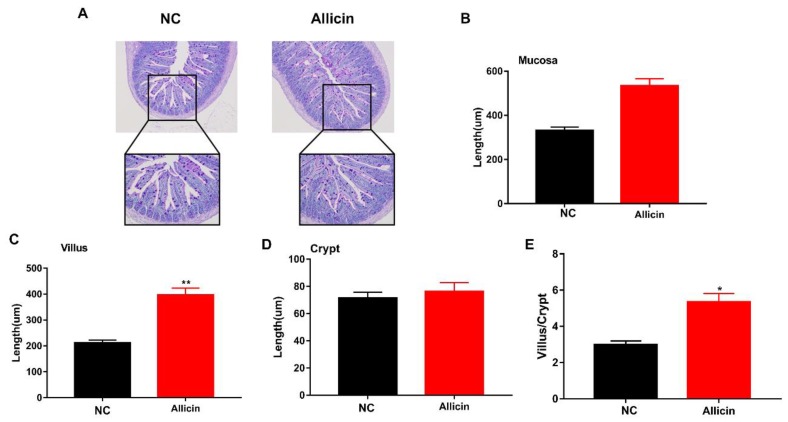
Improved intestinal morphology in obese mice induced by high-fat diet. (**A**) H&E staining of the small intestine from representative mice of each group. Images are shown at the original magnification of 200 (*n* = 3). (**B**) The mucosal thickness was measured in the H&E staining images in A. (**C**) The villus length was measured in the H&E staining images in A. (**D**) The crypt depth was measured in the H&E staining images in A. (**E**) The ratio of villus length to crypt depth is based on the measurements in C and D. The values represent the mean ± SD. * *p* < 0.05 and ** *p* < 0.01; *n* = 6.

Obesity is often associated with inflammation. Many studies have shown that intestinal morphology is strongly associated with fat deposition and inflammation. Intestinal villi and crypts directly reflect intestinal morphology. Previous studies have shown that the length of the villus and the depth of the crypt directly determines the nutrient absorption efficiency of the small intestine [21,22]. To explore the effect of allicin on intestinal morphology in mice with high-fat diet-induced obesity, we performed H&E staining on the jejunum. Allicin improved the intestinal morphology of obese mice (Figure 3A). Next, we measured the mucosal thickness, villus length and crypt depth in the small intestine in the images and found that allicin had no effect on mucosal thickness and crypt depth (*p* > 0.05; Figure 3B–D). However, it significantly increased villus length and the ratio of villus length to crypt depth (*p* < 0.05; Figure 3E). These results indicated that allicin improved intestinal morphology by changing the ratio of intestinal villus length to crypt depth.

### 3.5. Allicin Improves Intestinal Enzymatic Activity in Obese Mice Induced by High-Fat Diet

**Figure 4:**
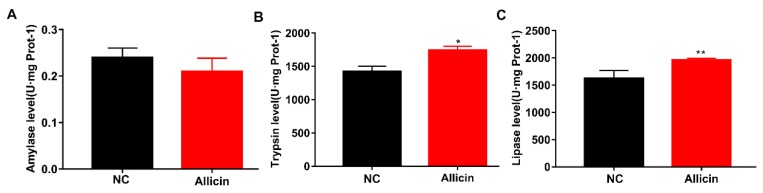
Improved intestinal enzymatic activity in mice with high-fat diet-induced obesity. (**A**) Amylase activity in the small intestine. (**B**) Trypsin activity in the small intestine. (**C**) Lipase activity in the small intestine. The values represent the mean ± SD. * *p* < 0.05 and ** *p* < 0.01; *n* = 6.

Intestinal villi secrete large amounts of enzymes to digest intestinal nutrients, which in turn affect intestinal digestion and absorption. To further investigate the effects of allicin on the intestine of obese mice, we examined the enzymatic activity of trypsin, amylase and lipase from the intestinal contents of the mice. Allicin significantly increased the enzymatic activity of trypsin and lipase (*p* < 0.05 and *p* < 0.01, respectively; Figure 4B,C), but had no effect on the enzymatic activity of amylase (*p* > 0.05; Figure 4A). These results indicated that allicin increased the absorption of nutrients in the small intestine by increasing intestinal enzymatic activity.

### 3.6. Allicin Does Not Affect Intestinal Microbial Structure in Obese Mice Induced by High-Fat Diet

**Figure 5:**
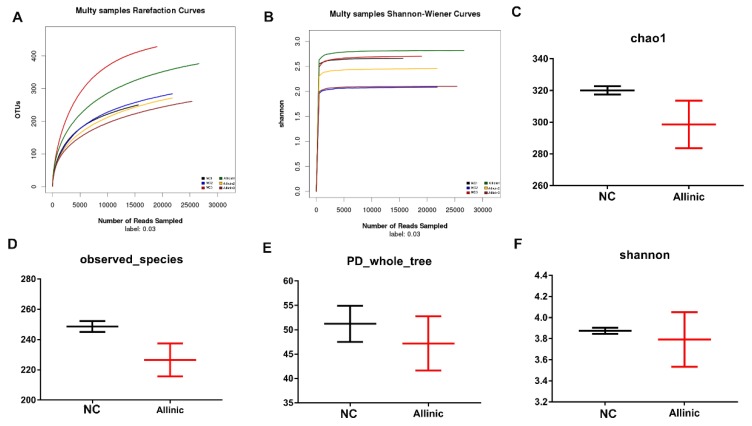
Allicin did not affect the intestinal microbial structure in obese mice induced by high-fat diet. (**A**) Rarefaction curves of the gut microbiota (GM). (**B**) Shannon index of the GM. (**C**–**F**) The α-diversity and the observed species of the GM.

Intestinal microorganisms are parasites that are affected by intestinal structure and enzymatic activity. Allicin affected intestinal morphology and enzymatic activity. To examine the effect of allicin on the regulation of the gut microbiota structure, high-throughput pyrosequencing was performed with an Illumina MiSeq platform. We generated 146,585 high-quality and valid sequences from six intestinal content samples from the different groups. The NC group produced 21,974 ± 1750 sequences per sample and the allicin group produced 26,888 ± 1548 sequences per sample. Based on a 97% similarity level, all of the effective reads were clustered into OTUs. The rarefaction curves indicated that although new species were obtained with the increase in the depth of sequencing, the current sequencing depth already contained most of the gut microorganism diversity (Figure 5A). The Shannon–Wiener curves showed that as the depth of sequencing increased, the curve reached a plateau, indicating that the current sequencing depth reflects most of the microbial information (Figure 5B). There were no significant differences in species abundance and diversity as judged by analysis of the chao1 (Figure 5C), observed species (Figure 5D), PD- whole tree (Figure 5E) and Shannon (Figure 5F) indices between the NC and allicin groups. These results indicate that allicin does not affect the structure of the intestinal microbiota.

### 3.7. Allicin Alters the Gut Microbiota in Mice with High-Fat Diet-Induced Obesity

**Figure 6:**
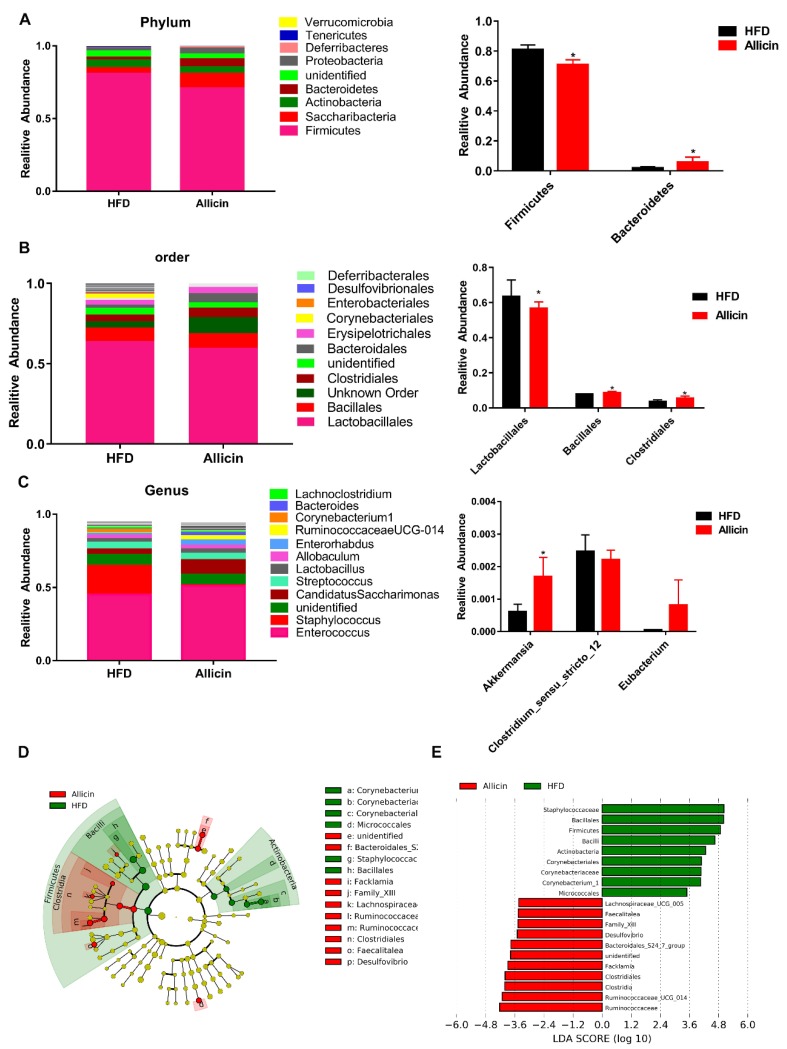
Alterations in the gut microbiota structure by allicin in mice with high-fat diet-induced obesity. (**A**) Composition analysis of the gut microbiota at the phylum level among all samples (left). The right panel shows the statistical analysis of the differences in microbiota at the phylum level. (**B**) Composition analysis of the gut microbiota at the order level among all samples (left). The right panel shows the statistical analysis of the difference in the microbiota at the order level. (**C**) Composition analysis of the gut microbiota at the genus level among all samples (left). The right panel shows the statistical analysis of the difference in the microbiota at the genus level. (**D**) Taxonomic cladogram obtained from linear discriminant analysis effect size (LEfSe) sequence analysis. Biomarker taxa are highlighted by colored circles and shaded areas. Each circle’s diameter reflects the abundance of that taxa in the community. (**E**) The taxa for which abundance differed between the HFD and allicin groups are indicated. HFD: mice fed a high-fat diet (*n* = 3); Allicin: mice fed a high-fat diet and received allicin (100 mg/kg; *n* = 3) for 8 weeks continuously. The values represent the mean ± SD. The cut-off value of ≥2.0 used for the linear discriminant analysis (LDA) in shown. * *p* < 0.05 and ** *p* < 0.01.

After allicin treatment, intestinal morphology and enzymatic activity in the mice changed, but the intestinal microbial structure did not change. To further explore the effects of allicin on intestinal microbial species, the gut microbiota was analyzed by 16S rDNA pyrosequencing at the phylum, order and genus levels (Figure 6A–C). At the phylum level, the Allicin group had a significantly decreased abundance of Firmicutes, but a markedly increased abundance of Bacteroidetes (Figure 6A). At the order level, allicin significantly increased the abundance of Bacteroidales and Clostridiales (Figure 6B). At the genus level, allicin significantly increased the abundance of *Akkermansia* (Figure 6C). To further clarify the changes in the intestinal microbiota at different levels, we compared the gut microbiota of the NC group and allicin group using the linear discriminant analysis (LDA) effect size (LEfSe) method. A cladogram representative of the structure of the gut microbiota and the predominant bacteria is shown in Figure 6D. The greatest difference in taxa between the two communities is displayed; allicin significantly elevated the relative abundance of Ruminococcaceae, Clostridiales, Bacteroidales and Facklamiaets, while it reduced the relative abundance of Firmicutes, Corynebacteriales and Lactobacillales (Figure 6E).

In summary, our results showed that allicin caused no significant differences in species abundance and diversity, but it highly shaped the gut microbiota in HFD mice; it increased the abundance of the Bacteroidetes phylum and decreased the abundance of the Firmicutes phylum.

## 4. Discussion

Studies have shown that the gut microbiota is an important environmental factor that contributes to the development of obesity, insulin resistance and inflammation [22]. The gut microbiota also promotes energy storage by suppressing thermogenesis in brown adipose tissues and promoting WAT expansion. Intestinal microorganisms produce many small, soluble metabolites, such as lipopolysaccharide, that induce proinflammatory cytokines, insulin resistance and WAT inflammation. These substances are absorbed by the intestines, transported to tissues and organs through the blood, and modulate metabolism and inflammation by regulating gene expression [23]. Allicin enhances insulin activity [24], and reduces hypertension and hyperlipidemia in diabetic patients [25]. Herein, we found that allicin decreased body weight gain and fat accumulation by inhibiting adipogenesis and promoting lipolysis. The GTT and RT-qPCR results of the insulin signaling pathway-related genes, *IRS1*, *IRS2* and *IRS3*, suggested that allicin significantly enhanced insulin sensitivity. Emerging evidence has demonstrated that allicin induces white adipocyte browning and reduces high-fat-induced obesity via the KLF15 signaling cascade [13,26]. In this study, we found that allicin increased the expression of brown adipocyte-related genes and thermogenic genes. These results suggest that allicin reduced the body weight gain of obese mice through white adipose browning.

Obesity is accompanied by an expansion in the volume of adipose tissues, causing an increase in mechanical stress by contact with neighboring cells and extracellular matrix components. When adipocytes spread to near the oxygen diffusion limit, they experience hypoxia, resulting in dysregulation of adipokine production and inflammation [27]. Under these circumstances, the secretion of leptin and resistin increases. In the current study, allicin reduced the expression of the adipokine-related genes, leptin, adipoq and resistin, and the area of fat cells decreased. These results indicated that allicin reduced fat deposition and inhibited inflammation in obese mice. Furthermore, we found that allicin reduced the LDL-C level in obese mice and increased the HLD-C and TG levels, which is consistent with previous research [12,28].

The intestine morphology directly reflects the health state of the intestine [29], which is mainly determined by the villi length, the crypt depth and their ratio. It is generally accepted that the ratio of villus to crypt is large, which is more conducive to the absorption of nutrients [30]. In this study, allicin significantly increased the villi length and the ratio of villus length to crypt depth, demonstrating that allicin improved intestinal function. It has been shown that intestinal microorganisms affect intestinal morphology [31], for example, the cecal *Bacillus fragilis* population negatively correlated with crypt depth, while the abundance of cecal C. leptum positively correlated with the villus height [32]. This indicated that allicin may affect intestinal morphology through gut microorganisms.

Recent research has revealed that a variety of small molecules substances, such as *Cordycepin*, *Fuzhuan brick tea* polysaccharides and *Vanillin*, can reduce fat deposition by altering intestinal microbial composition [6,33,34]. However, allicin-induced reduction in body weight gain of high-fat diet-induced obese mice through the gut microbiota has not been reported. In this study, allicin affected the intestinal microbial composition in obese mice induced by high-fat diet.

Gut microorganisms perform many functions that the body cannot perform, hence, they form a symbiotic relationship with the body. In 1983, Wostmann first discovered that in a germ-free (GF) environment, the rate of weight gain was slower than in a normal environment [35]. Bäckhed et al. have further demonstrated that intestinal microorganisms suppressed the intestinal expression of angiopoietin-like 4 (ANGPTL4), a circulating inhibitor of lipoprotein lipase (LPL). This increased the LPL activity in adipocytes, then increased the absorption of fatty acids by the cells and the accumulation of TG in the adipocytes [36]. Under GF conditions, the expression level of ANGPTL4 was higher, and LPL was inhibited, resulting in a slower rate of weight gain in mice. These findings showed that the intestinal microbiota plays an important role in fat formation.

In the intestinal microbiota, Firmicutes, Bacteroidetes and Actinobacteria account for more than 90% of all of the bacteria. It has been shown that obese mice were associated with gut microbiota changes; in ob/ob mice, the abundance of Firmicutes was increased, while the Bacteroidetes abundance was decreased. In contrast, in lean mice, the Firmicutes abundance was decreased, while the Bacteroidetes abundance was increased [37]. This is consistent with the intestinal microbial differences observed between lean and obese humans [6]. Our research revealed that allicin significantly increased the abundance of Bacteroidetes and decreased the abundance of Firmicutes in obese mice induced by a high-fat diet. This indicates that allicin affects intestinal microbial composition at the phylum level. Thus, the ratio of Firmicutes and Bacteroidetes can be used as a biomarker for obesity. However, several studies have found that the Firmicutes and Bacteroidetes ratio increased in obese individuals [38,39]. Hence, a lower level analysis is necessary to detect changes in gut microorganisms. Therefore, we chose to use the order and a lower level of genus for comparison and analysis.

At the order level, Lactobacillus and Bifidobacterium are typically used as beneficial bacteria, and studies have found that the abundance of both was increased in obese individuals [40]. In this study, we found that Bifidobacterium was significantly increased in allicin-treated mice. This was consistent with the findings of Lecomte et al. [41]. Namely, the abundance of two bacterial species in obese mice depends on the experimental model. At the genus level, we analyzed the abundance of *Akkermansia*, *Clostridium* XIVb and *Eubacterium*.

Studies have shown that *Akkermansia* colonizes the mucosal layer of the human intestine and increases mucosal thickness and gut barrier function. *Akkermansia* also sends a signal directly to immune receptors, causing a host immune response, and at the same time it produces short-chain fatty acids that are beneficial for the host and for microbiota members [42]. *Clostridium* XIVb and *Eubacterium* had an anti-obesity effect by producing butyrate, which is a source of energy for the colonocytes [43]. Our results showed that at the genus level allicin significantly increased the abundance of *Akkermansia*; however, it had no effect on *Clostridium* XIVb and *Eubacterium*. These results indicated that allicin reduced body weight gain and fat accumulation by increasing the abundance of beneficial species [5].

Allicin has been widely studied for its anti-inflammatory, anti-cancer, anti-hypertensive and anti-obesity effects [44]. Other study have found that allicin can alleviate the learning and memory disorders caused by exposure to lead during development [45]. Interestingly, Cai et al. found that *Flammulina velutipes* polysaccharides improved scopolamine-induced learning and memory impairment in mice by regulating the composition of intestinal microbes [46]. In this study, we found that allicin can induce weight loss in obese mice induced by high-fat diet by regulating intestinal microbes. Therefore, we suspect that the improvement of memory and learning ability of allicin may also be achieved by intestinal microbes. But further research is needed.

## 5. Conclusions

**Figure 7:**
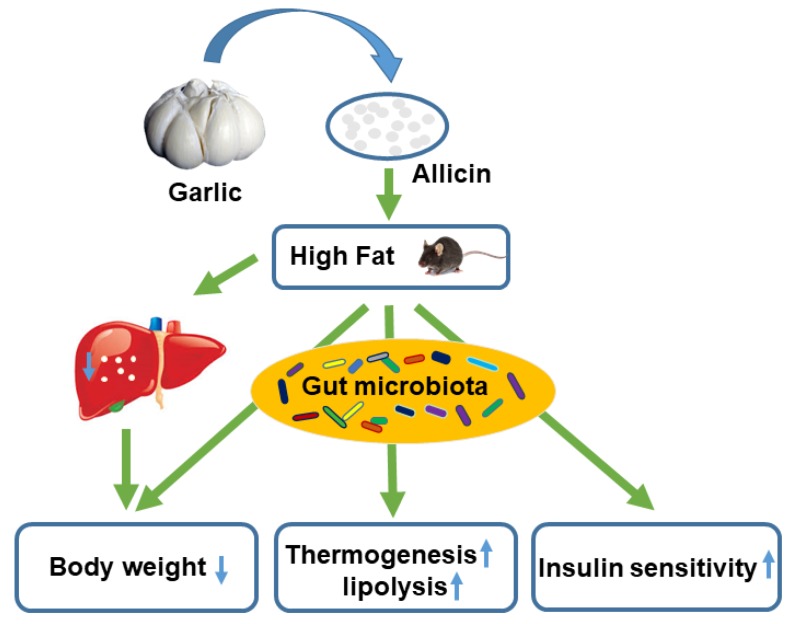
Summary diagram showing that via the gut microbiota allicin may affect the body weight, lipolysis, thermogenesis and insulin sensitivity of obese mice induced by a high-fat diet.

We believe that allicin improves metabolism in high-fat diet-induced obese mice by regulating the composition of the intestinal microbiota (Figure 7), which provides a potential theoretical basis for the treatment of obesity by allicin.

## Figures and Tables

**Table 1:** Allicin ameliorated blood metabolic parameters in mice with high-fat diet-induced obesity.

Parameter	NC	Allicin	*p*-Value
AST, U/L	246.64 ± 139.77	196.62 ± 34.18	0.4593
ALT, U/L	43.86 ± 17.58	50.12 ± 11.34	0.5223
TC, mmol/L	2.59 ± 0.45	3.67 ± 0.51	0.0076
TG, mmol/L	0.82 ± 0. 20	0.91 ± 0.11	0.4077
HDL-C, mmol/L	1.40 ± 0.16	2.07 ± 0.18	0.0003
LDL-C, mmol/L	0.33 ± 0.05	0.26 ± 0.06	0.0808

Values are expressed as the mean ± SEM of five animals (*T*-test). Abbreviations: AST, aspartate amino transaminase; ALT, alanine amino transaminase; TC, serum cholesterol; TG, serum triglycerides; HDL-C, high-density lipoprotein (HDL)-cholesterol; LDL-C, low-density lipoprotein (LDL)-cholesterol.

## Supplementary Materials

The following are available online at https://www.mdpi.com/2072-6643/11/12/2909/s1, Table S1: Primer sequences.
