# Supplementary material for: Allicin Improves Metabolism in High-Fat Diet-Induced Obese Mice by Modulating the Gut Microbiota
Source: Nutrients. 2019 Dec 2;11(12):2909. doi: 10.3390/nu11122909 (PMC6949904; doi:10.3390/nu11122909)
Supplement: Supplementary file 1 [file nutrients-11-02909-s001.pdf]

**Genes**	**Primers**	**Nucleotide sequence of primers (5'-3')**	**Size(bp)**
ATGL	Forward	TTCGCAATCTCTACCGCCTC	136
	Reverse	AAAGGGTTGGGTTGGTTCAG	
HSL	Forward	GCTGGGCTGTCAAGCACTGT	160
	Reverse	GTAACTGGGTAGGCTGCCAT	
LPL	Forward	CCAATGGAGGCACTTTCCA	85
	Reverse	TGGTCCACGTCTCCGAGTC	
PGC1a	Forward	AGCCGTGACCACTGACAACGAG	168
	Reverse	GCTGCATGGTTCTGAGTGCTAAG	
UCP1	Forward	GGCATTCAGAGGCAAATCAGCT	151
	Reverse	CAATGAACACTGCCACACCTC	
CIDEA	Forward	TGCTCTTCTGTATCGCCCAGT	113
	Reverse	GCCGTGTTAAGGAATCTGCTG	
Cox7a	Forward	GCTCTGGTCCGGTCTTTTAGC	100
	Reverse	GTACTGGGAGGTCATTGTCGG	
PRDM16	Forward	CGGAAGAGCGTGAGTACAAATG	133
	Reverse	TCCGTGAACACCTTGACACAGT	
Adipoq	Forward	GTCTGTACGATTGTCAGTGGATCTG	78
	Reverse	AAGAGGAACAGGAGAGCTTGCA	
Leptin	Forward	GAGACCCCTGTGTCGGTTC	139
	Reverse	CTGCGTGTGTGAAATGTCATTG	
Resistin	Forward	AAGAACCTTTCATTTCCCCTCCT	167
	Reverse	GTCCAGCAATTTAAGCCAATGTT	
IRS1	Forward	CGATGGCTTCTCAGACGTG	209
	Reverse	CAGCCCGCTTGTTGATGTTG	
IRS2	Forward	CTGCGTCCTCTCCCAAAGTG	124
	Reverse	GGGGTCATGGGCATGTAGC	
IRS3	Forward	TCGGCTCACCGTTTCCTTG	133
	Reverse	TCGCTCTCGTAGCACTCCA	
